# A single birth dose of Hepatitis B vaccine induces polyfunctional CD4^+^ T helper cells

**DOI:** 10.3389/fimmu.2022.1043375

**Published:** 2022-11-08

**Authors:** Julia Strandmark, Alansana Darboe, Joann Diray-Arce, Rym Ben-Othman, Sofia M. Vignolo, Shun Rao, Kinga K. Smolen, Geert Leroux-Roels, Olubukola T. Idoko, Guzmán Sanchez-Schmitz, Al Ozonoff, Ofer Levy, Tobias R. Kollmann, Arnaud Marchant, Beate Kampmann

**Affiliations:** ^1^ Vaccines & Immunity Theme, Medical Research Council (MRC) Unit The Gambia at London School of Hygiene & Tropical Medicine (LSHTM), Fajara, Gambia; ^2^ Precision Vaccines Program, Boston Children’s Hospital, Boston, MA, United States; ^3^ Department of Pediatrics, Harvard Medical School, Boston, MA, United States; ^4^ Department of Paediatrics, University of British Columbia, Vancouver, BC, Canada; ^5^ Center for Vaccinology, Ghent University Hospital, Ghent, Belgium; ^6^ Klarman Cell Observatory & Global Health Initiative, Broad Institute of the Massachusetts Institute of Technology (MIT) & Harvard, Cambridge, MA, United States; ^7^ Institute for Medical Immunology, Université Libre de Bruxelles, Brussels, Belgium; ^8^ The Vaccine Centre, London School of Hygiene and Tropical Medicine, London, United Kingdom

**Keywords:** CD4 + T helper cell, T-cell activation, CD154, Hepatitis B vaccine, BCG, antibody titres, neonate

## Abstract

A single birth-dose of Hepatitis B vaccine (HepB) can protect newborns from acquiring Hepatitis B infection through vertical transmission, though several follow-up doses are required to induce long-lived protection. In addition to stimulating antibodies, a birth-dose of HepB might also induce polyfunctional CD4^+^ T-cells, which may contribute to initial protection. We investigated whether vaccination with HepB in the first week of life induced detectable antigen-specific CD4^+^ T-cells after only a single dose and following completion of the entire HepB vaccine schedule (3 doses). Using HBsAg- stimulated peripheral blood mononuclear cells from 344 infants, we detected increased populations of antigen-specific polyfunctional CD154^+^IL-2^+^TNFα^+^ CD4^+^ T-cells following a single birth-dose of HepB in a proportion of infants. Frequencies of polyfunctional T-cells increased following the completion of the HepB schedule but increases in the proportion of responders as compared to following only one dose was marginal. Polyfunctional T-cells correlated positively with serum antibody titres following the birth dose (day30) and completion of the 3-dose primary HepB vaccine series (day 128). These data indicate that a single birth dose of HepB provides immune priming for both antigen-specific B- and T cells

## Introduction

The infant immune system differs from that of older children and adults, including reduced ability to mount T-helper 1 (Th1) responses or sustain high antibody titres, leading to potentially suboptimal induction of immunity to certain vaccines administered at birth (1, 2). Accordingly, most vaccines are delivered several months after birth, leaving young infants vulnerable to infection in early infancy. The infant immune system is however not inherently inert, as exposure to some antigens, such as the live attenuated *Mycobacterium bovis* vaccine, Bacille Calmette–Guérin (BCG), stimulate immune responses at least comparable to those seen in adults (3). Moreover, BCG induces a range of non-specific immune functions that lead to reduced overall mortality, particularly when given in the neonatal period (4). The subunit Hepatitis B vaccine (HepB), containing surface antigens from the Hepatitis B virus (HBV), is immunogenic in newborns although long-lasting immunity, measured as sustained high levels of HepB specific antibodies, requires repeated doses over several months.

Although neutralizing antibody is the recognised mechanism through which protection against HBV is mediated, data suggest that other immune responses, e.g. vaccine-induced CD4^+^ T- helper cell responses might also contribute to protection (5, 6). For example a portion of vaccinated individuals are known to mount insufficient antibody levels (<10IU/l), despite apparent clinical protection (5, 7) and T-cell memory persists beyond the waning of antibody titres to levels below 10IU/l (5, 6, 8). Furthermore, a birth-dose of HepB reduces up to 75% of vertical HepB transmission from mother to child (9)- the most common route of transmission for HBV (10). Considering that several subsequent doses are required to induce seroconversion in most infants, this suggests that alternative immune pathways might contribute to initial protection in the newborn.

With few exceptions (11), most studies reporting on cell mediated immunity induced by HepB were conducted in adults or older children, with little to no data on immunity induced by the first neonatal dose. Studying T-cell responses in very young infants is challenging due to small blood volumes, relatively low frequencies of antigen-specific T-cells and the possibility that markers of activation used in adults may be unsuitable for infant T-cells (12–14). Nevertheless, data in very young infants will provide insight into how vaccines induce immune responses in a vulnerable population that may respond poorly to antigenic stimulation and may thus be of use in our efforts to close the gap of vulnerability early in life.

Here we investigated whether vaccination against HepB in the first week of life results in induction of antigen-specific polyfunctional CD4^+^ T-cells. We further asked whether HepB specific CD4^+^ T-cells increase with the completion of a full HepB vaccine schedule and whether there is a correlation of this response with antibody titres to Hep B surface antigen (HBsAb). We used multicolour flow cytometry and the expression of the activation marker CD154, as well as IL-2 and TNFα to determine whether antigen- specific CD4^+^ T-cells can be detected in infants at day 30 (D30) and day 128 (D128) of life- one month after primary HepB vaccination or following completion of the vaccine schedule respectively.

## Materials and methods

### Study participants and vaccination schedule

The study was nested within the Expanded Programme of Immunization Consortium-Human Immunology Project Consortium (EPIC-HIPC) study (https://clinicaltrials.gov/ct2/show/NCT03246230), in which 720 term newborns were enrolled at Kanifing General Hospital and Banjulinding Health Centre in the Western Region of The Gambia. As part of this study, some infants were vaccinated at birth with HepB (HepatitisB subunit vaccine, Serum Institute of India) and BCG (Bacille Calmette–Guérin, live attenuate vaccine, Serum Institute of India), whereas others were initially given either HepB or BCG at birth, with the missing vaccine being administrated on D1, -3, or -7 following blood collection. All infants receiving vaccinations within the first week of life were invited for follow-up visit on D30 (one month post primary vaccination) and D128 (1 month post completion of the last dose of HepB) and were included in this sub-study (n = 540). The full study protocol outlining maternal inclusion/exclusion criteria, informed consent and recruitment procedures has been previously published (15), as has details of data management (16).

Across the entire study period, cryopreserved cells from adult donors were included to serve as comparison to infant immune responses. All donors (n=3) were healthy, Gambian males with an average age of 35 years, who had been vaccinated with HepB within the last 7 years.

### PBMC separation

3ml of peripheral venous blood was drawn *via* sterile venepuncture directly into heparinized collection tubes (BD Biosciences; CA, USA) and brought to the Medical Research Council Unit The Gambia at LSHTM in Fajara for isolation of peripheral blood mononuclear cells (PBMCs) within 4 hours. Samples were centrifuged at 2000 rounds per minute (720g) to separate plasma, which was aliquoted and frozen at -80°C for subsequent anti-HBs antibody titre analysis. The remaining cells were resuspended in R0: RPMI-1640, containing 1% L-Glutamine (both from Sigma-Aldrich, UK), 100U/ml Penicillin and 100 µg/ml Streptomycin (Sigma-Aldrich, Israel), 0.5mM Sodium Pyruvate and 6mM HEPES with 3% Foetal Bovine Serum (FBS) (all from Gibco, Germany). The diluted cells were layered over Ficoll-paque (GE Healthcare, Sweden) at a ratio of 2:1 (diluted blood: Ficoll-paque) and spun at 2400 rpm (1037g) for 30 minutes without brake. The separated PBMCs were washed twice and resuspended in complete medium: R0 with 10% FBS. Cells were counted using a Luna II automated cell counter (Logos Biosystems, South Korea).

PBMCs from adult donors were separated as above, aliquoted at 10x10^6^ cells/ml and frozen in freezing media (10% DMSO in FBS) at -80°C. Thawed cells were included in 90/111 flow cytometry runs and replenished as needed.

### PBMC stimulation

2 x 10^6^ (BCG and HBsAg) or 0.5x10^6^ (SEB) PBMCs in a final volume of 500µl were stimulated in polypropylene tubes (Falcon Corning^®^, USA) with 1µg/ml Hepatitis B surface antigen pooled peptide (HBsAg) (17), 10-40x10^4^ CFU/ml BCG, 1µg/ml SEB antigen (Sigma-Aldrich, UK) or complete RPMI and incubated at 37°C for 18 hours at 5% CO_2_ (see **Table S1** for calculation of antigen concentrations). Anti-human CCR7- PECF594 antibody (R&D systems, USA) was added to each tube at the start of the culture and 5µg/ml Brefeldin A (Sigma-Aldrich, UK) was added 2 hours after start, to prevent cytokine release. Inclusion of BCG stimulation commenced ~2,5 months after initial study start.

### Flow cytometry

Following 18hr stimulation, PBMCs were washed in FACS buffer: PBS supplemented with 2mM EDTA and 0.1% Bovine Serum Albumin (all from Sigma-Aldrich, UK), and stained with the viability marker LIVE/DEAD™ (Invitrogen™) followed by surface staining, using the following conjugated anti-human antibodies: CD4-V450, CD3-V500 (both from BD Bioscience), CD27-PE-Cy7 and CD45RA-FITC (both from eBioscience). (See **Table S2** for full details of antibodies and the dilutions used). Cells were incubated in the dark, for 20 minutes at 4°C. Once staining of surface markers was completed, samples were fixed in 150µl of Cytofix/Cytoperm solution (BD Cytofix/Cytoperm™) for 15 minutes at 4°C, after which cells were washed twice in 1 x Perm Wash buffer (BD Perm/Wash™). Intracellular staining employed the following conjugated antibodies: CD154-APC-Cy7, IL-2-APC (both from BioLegend^®^) and TNFα-AF700 (BD Bioscience). Cells were incubated in the dark for 30 minutes at 4°C. After washing in Perm Wash buffer, cells were acquired on a BD LSRFortessa™ Flow Cytometer and analysed with DB FACSDiva™ software. Infant cells were assayed fresh in a total of 111 assay runs over the 2 year study period. On each acquisition day, UltraComp eBeads™ (Invitrogen) were prepared as single stained controls and unstimulated samples were included for each participant. FCS files were analysed with FlowJo (TreeStar, USA). A template gating strategy was created based on fluorescent minus one samples and used to analyse all FCS files. Boolean gating analysis was applied to identify subsets expressing combinations of activation markers (18).

Markers for detection of antigen-specific memory T-cells (CD45RA, CCR7, CD27) were included in panel, but excluded from analysis, as cell populations in HepB stimulated samples were consistently too small to reliably detect.

### Determination of anti-HBs antibody titres

Anti-HBs antibody (HBsAb) titres were measured at the Centre for Vaccinology, Ghent University Hospital, Belgium, using an Architect analyser (Abbott Laboratories; Chicago, IL, USA) according to the manufacturer’s instructions (kit reference 7C18). The analytical range of the HBsAb assay is 2.50 – 1000 mIU/mL. Negative and positive assay controls were included in each run as provided by the manufacturer. In the case of insufficient sample volume, a dilution was made using Fetal Calf Serum (FCS). Sample with low volume of < 10 µL were not tested and were reported as Quantity Not Sufficient (QNS). Samples with a titre > 1000 mIU/mL were manually diluted to 1:100 with FCS as per manufacturer instruction and retested. Samples with a titer below the lower limit of the analytical range (2.5 mIU/mL) were reported as < 2.50 mIU/mL.

To avoid interference from transferred maternal antibodies when performing correlation analysis between cell mediated immunity (CMI) and antibody titres, only infants in whom anti- HBsAb titres were undetectable at birth were included in the correlational analysis.

### Data analysis and statistics

Cell frequency data were analysed using RStudio ServerPro and visualized using Prism GraphPad. A set of quality control criteria (**Table S3)** were applied and samples that did not meet these criteria were excluded from the analysis. T-cell activation on D30 following antigen stimulation was initially compared to background (unstimulated sample) activation (**Figure 2**). Before comparing T-cell activation between D30 and D128, background activation from unstimulated samples was subtracted from matched antigen-stimulated sample (**Figures 3**, **4**). In this case, zero and negative values were assigned a value of 0.001 and all values were log10- transformed for visualization.

Antibody titres reported as below the analytical range were assigned the value of 2.5 (lower limit of analytical range) and all values were log10 transformed.

Kruskal-Wallis one way analysis of variance with Dunn’s multiple comparison enabled comparison of the activation of CD4^+^ T helper cells between stimulation conditions and time-points. Responders to antigen stimulation were defined as having T-cell frequencies above 2 times unstimulated sample.

Spearman’s correlation analysis between cell mediated immunity and antibody titres was performed on log10 CMI responses versus log10 antibody titres of the same participants.

A p-value of <0,05 was considered statistically significant.

## Results

### Study sample

**Figure 1:**
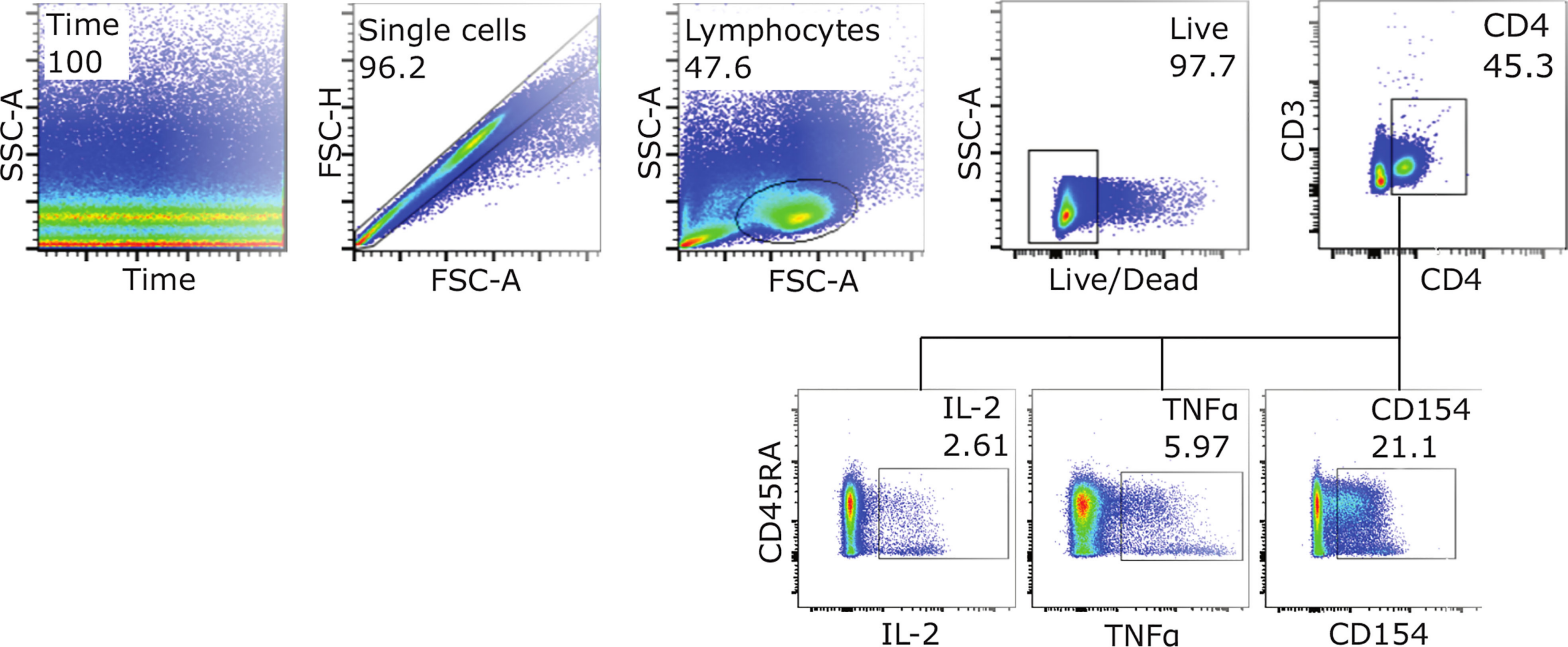
Gating strategy for detection of activated CD4^+^ T-cells. Example FACS plots from SEB stimulated infant PBMSs at D128, showing the gating strategy used to measure the expression of activation markers IL-2, TNFα and CD154 (CD40L). To assess frequencies of cells expressing all three markers, Boolean gating was applied.

540 infant study participants completed the per protocol visits on D30 and D128 of life, from whom 518 (D30) and 509 (D128) samples were successfully collected and analysed for T-cell activation by flow cytometry (**Figure 1**). In total, 174 (D30) and 165 (D128) samples were subsequently excluded due to pre-determined exclusion criteria (**Table S3**). Finally, 344 HBsAg-, 254 BCG- and 328 SEB- stimulated, matched (D30 and D128) samples were available for the analysis of T-cell responses. Sex, average birthweight, and average day of sampling of the population included in the final sample is outlined in **Table S4**. Cryopreserved PBMCs from 3 adult donors were included in a total of 90 (out of 111) assay runs. Following application of exclusion criteria, a total of 73 adult samples were included in analysis (**Table S4**).

### HepB specific CD4^+^ T-cells can be detected in neonates after single dose of HepB vaccine

**Figure 2:**
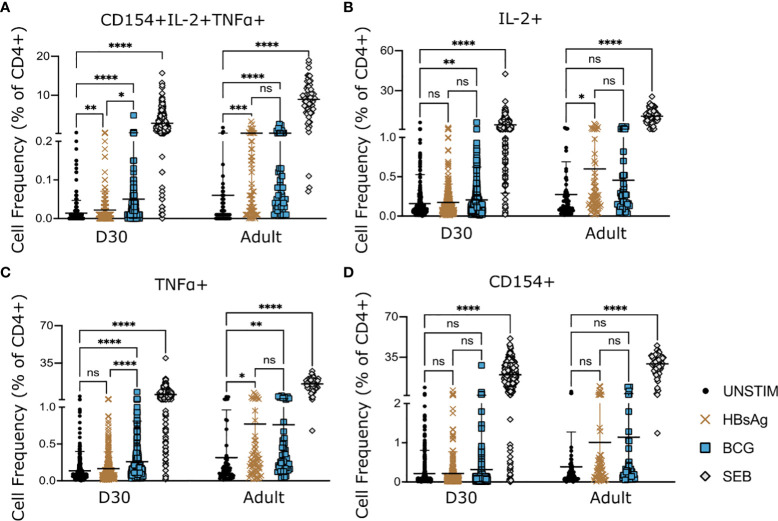
HepB- and BCG-specific CD4^+^ T-cells in infant and adult samples. PBMCs from infants at D30 and adult donors (pooled data from 3 donors) were stimulated with HBsAg, BCG or SEB for 18hrs. Expression of activation markers CD154, IL-2 and TNFα, following stimulation was compared to background (unstimulated samples). Cell frequencies were analysed based on expression of all three markers **(A)** or total expression of one marker **(B–D)**. Kruskal-Wallis with Dunn’s post test, *p < 0,05; **p < 0,01; *** p < 0,005; **** p < 0,001; ns, not significant.

Thirty days post vaccination, HepB specific, polyfunctional CD4^+^ T-cells co-expressing IL-2, TNFα and CD154 could be detected in neonates having received only one dose of HepB vaccine in the first week of life (**Figure 2A** **).** In contrast, HepB specific T-cells could not be detected using the expression of IL-2, TNFα or CD154 alone (**Figures 2B–D** **).**

BCG specific T-cells could be detected on D30 post vaccination either through polyfunctional expression of all three markers or through total expression of IL-2 or TNFα, but not through CD154 expression (**Figures 2A–D**).

In adult samples, HBsAg-specific CD4^+^ T cells could be readily detected using either co-expression of all markers (**Figure 2A**) or total expression of IL-2 or TNFα (**Figures 2B, C**). Using CD154 alone did not detect a significant increase in HBsAg specific CD4^+^ T-cells in adult samples (**Figure 2D**).

We confirmed that the separation of BCG vaccine co-administration in the first week of life (see Materials & Methods) did not influence HepB specific T cell responses, by separating the data according to their vaccination groups. This analysis revealed no difference in HepB specific CD4^+^ T cell activation depending on timing of BCG co-administration during the first week of life (**Figure S2**), and thus justified the continued analysis of all vaccine groups together.

### Polyfunctional CD4^+^ T-cells increase with age and completion of HepB vaccination

We next examined whether HepB specific T-cells increase further with the completion of the HepB vaccine schedule (D128).

**Figure 3:**
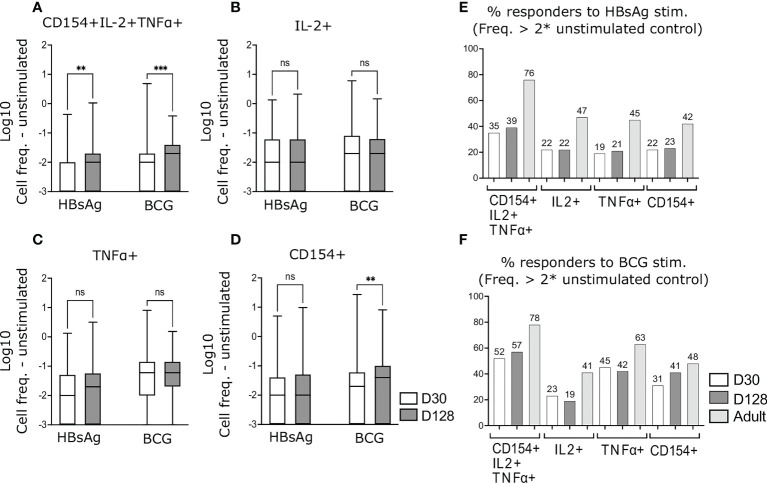
Antigen-specific CD4^+^ T-cells following completion of HepB vaccine schedule. PBMCs from infants at D30 and D128 were stimulated and stained as in **Figure 2**. Background activation was removed by subtracting values from unstimulated matched samples. Cell frequencies of CD4^+^ polyfunctional T-cells **(A)** or frequencies based on total expression of IL-2 **(B)**, TNFα **(C)** or CD154 **(D)** were compared. Responders to HBsAg **(E)** and BCG **(F)** stimulation were calculated as having cell frequencies above 2 x of the matched unstimulated sample. **(A–D)** Zero and negative values were assigned a value of 0.001 to enable Log transformation. Data are expressed as Log10 of CD4^+^ T-cell frequencies. Kruskal-Wallis with Dunn’s post-test, **p < 0,01; ***p < 0,005; ns, not significant.

Following the subtraction of background, significantly higher frequencies of HepB-specific, polyfunctional CD4^+^ T-cells, expressing all three activation markers, were detected in infants on D128, as compared to D30 (**Figure 3A**). However, using the more stringent criteria to define responders (see Materials and Methods), we detected an increase of only 4% from 35% at D30 to 39% at D128 (**Figure 3E**).

Using the expression of one activation marker only (IL-2, TNFα or CD154) following HBsAg stimulation, activated cells did not increase by D128 as compared to D30 (**Figures 3B–D**) and the proportion of infants responding to stimulation did not change (**Figure 3E**).

Similar to HBsAg-specific cells, frequencies of polyfunctional BCG- specific CD4^+^ T-cells, expressing all three markers significantly increased at D128 as compared to D30 (**Figure 3A**), whereas frequencies of BCG specific IL-2^+^ and TNFα^+^ cells remained comparable in both age groups (**Figure 3B, C**). In contrast, frequencies of BCG-specific cells expressing CD154 on its own increased in D128 old infants, as compared to D30 (**Figure 3D**). A higher proportion of infants responded to BCG stimulation as compared to HBsAg stimulation and the highest proportion of BCG responders were detected by measuring polyfunctional cells (**Figure 3F**).

### Polyfunctional HepB specific CD4^+^ T-cells correlate with anti-HBs antibodies

We next examined whether HepB specific T-cell responses correlate with anti-HBsAb titres in young infants.

At D30, 18% of included infant samples had antibody titres above 10IU/l, whereas all infants had seroconverted by D128 (**Figure S3**). All populations of activated CD4^+^ T-cells could be detected in a proportion of infants in with antibody titres were below 10IU/l at D30. A higher frequency of polyfunctional CD4^+^ T-cells were detected in infant with HBsAb titres >10 IU/l compared to infants with titres < 10IU/l (**Figure S3**).

**Figure 4:**
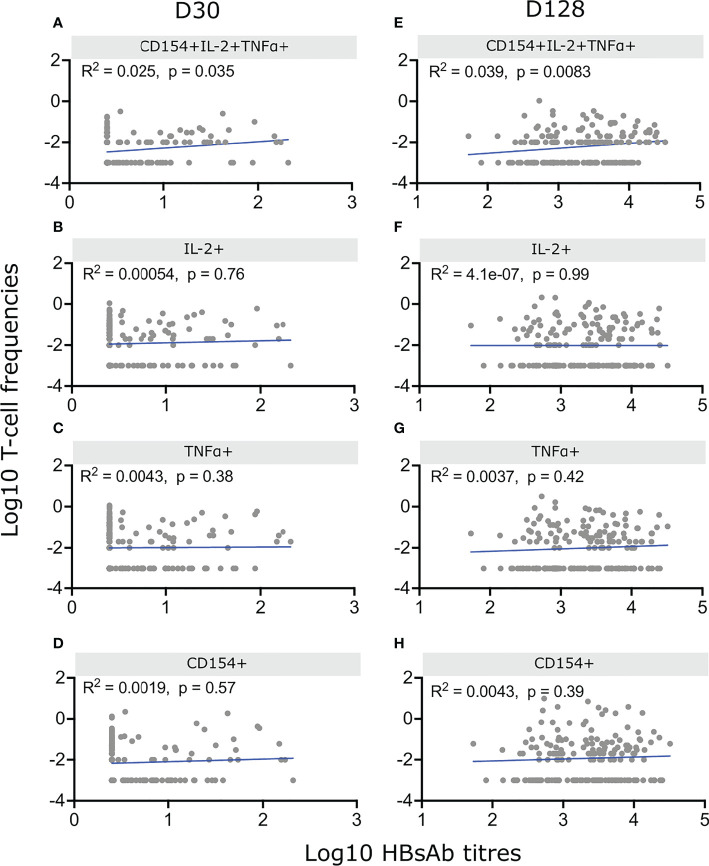
Correlation between HepB specific CD4^+^ T-cells and antibody titres. Infants were vaccinated with HepB in the first week of life and received additional doses at 6, 10 and 14 weeks. IgG1 antibodies were measured at D30 and D128 and PBMCs from the same blood draws were stimulated with HBsAg for 18hrs and stained as in **Figures 2, 3**. Cell frequencies of CD4^+^ polyfunctional T-cells **(A, E)** or frequencies based on total expression of IL-2 **(B, F)**, TNFα **(C, G)** or CD154 **(D, H)** were correlated with their matched antibody titres on D30 **(A–D)** or D128 **(E–H)**. Background CD4^+^ T-cell activation was removed by subtracting values from unstimulated matched samples. Only infants with undetectable HepB-specific antibody titres at birth and available matched Ab – T-cell sample (n = 179) were included. Spearman correlation was performed on Log10 of CD4^+^ T-cell frequencies and Log10 of antibody titres. P < 0,05 was considered significant.

We performed correlation analysis between HBsAb titres and frequencies of HepB specific CD4^+^ T-cells and detected a significant correlation between polyfunctional CD154^+^IL-2^+^TNF^+^ CD4^+^ T-cells and anti-HBs antibody titres at D30 (R^2 =^ 0.025, p=0.035) and D128 (R^2^ = 0.039, p=0.0083) but no correlation between antibody titres and HepB specific CD4^+^ T-cells when measuring the expression of only one of the three activation markers (**Figure 4**).

## Discussion

We have demonstrated that (a) HepB specific, CD4^+^ T-cells could be detected in vaccinated infants after only a single birth dose of HepB vaccine; (b) frequencies of antigen specific polyfunctional (CD154^+^ IL-2^+^ TNFα^+^) CD4^+^ T-cells increased following the completion of a full, 3- dose HepB vaccination schedule; and (c) polyfunctional CD4^+^ T-cells correlated with anti-HBs antibody titres.

### Polyfunctional T-cells after birth-dose

By D30, polyfunctional CD4^+^ T-cells, expressing all three markers included in the study (CD154, IL-2 and TNFα) were the most prominently upregulated CD4^+^ cell population in neonates who received HepB vaccine at birth.

T-cells expressing more than one cytokine and/or co-stimulatory molecule at once have received much attention, as evidence accumulate suggesting that the quality of the T-cell response is likely more important in vaccine- induced protection, than the quantity of activated cells (19, 20). Polyfunctional T-cells produce more cytokine per cell than monofunctional cells (21, 22), have higher co-stimulatory and cytotoxic potential (21) and are more likely to survive as memory cells (18). Polyfunctional CD4^+^ T-cells correlate with protection against viral and parasitic diseases (20, 21) and CD4^+^ cells co-expressing IFNγ, TNFα and IL-2 have been explored as a potential correlate of vaccine-induced protection against *Mycobacterium tuberculosis* (22), though so far no conclusive link has been demonstrated. Based on these findings, it has been suggested that the induction of polyfunctional T-cells in particular, should be a target of novel vaccine candidates aiming to induce a cellular response (21). It was thus encouraging to see that polyfunctional CD4^+^ T-cells could be detected in neonates vaccinated with HepB in the first week of life. As the cellular response to HepB vaccination in neonates this young has only rarely been investigated (11), this adds novel insight into the early adaptive immune functions induced by this highly effective vaccine.

Maternal antibodies may inhibit infant cellular immune responses to some vaccine antigens (2). As our analysis of HepB specific CD4^+^ T-cells in neonates included all infants, regardless of the antibody status of the mother, it is possible that T-cell responses in some infants were suppressed due to maternally transferred antibody, potentially contributing to the observation that only a proportion of infants showed increases in HepB specific CD4^+^ T-cells. A more detailed analysis of the effect of maternally transferred antibodies on HepB induced immunity is ongoing.

Due to the overall study design, neonates in this study received HepB and BCG vaccinations staggered across the first week of life. It was thus important to investigate whether the separation of BCG from HepB by up to 7 days influenced the frequencies of HepB specific responses on D30 or on D128, in particular as BCG co-administration with HepB has previously been reported to enhance HepB specific immune responses (11, 23). However, we detected comparable levels of HBsAg activated CD4^+^ T-cells in all vaccine- randomisation groups, regardless of the order in which the vaccines were given. We therefore conclude that a priming effect of BCG (11, 23) either takes place even when the two vaccines are temporally separated within the first week of life, or that BCG had no effect on HepB antibody responses in our setting.The current setting did not allow to investigate this further, as withholding BCG vaccination to allow for an unvaccinated control group in a tuberculosis- endemic region such as The Gambia was not deemed ethical. Thus, further studies investigating the time-window in which BCG might enhance unrelated immune responses will be necessary to conclude whether co-administration of the vaccines impacts HepB CD4^+^ T-cell responses.

### Polyfunctional cells with age and completion of HepB

Our data show that though frequencies of polyfunctional CD4^+^ T-cells increased in neonates who had received their full HepB vaccination schedule, the proportion of infants classified as responders increased only marginally. This contrasted with humoral immunity, where the completion of the vaccination schedule resulted in protective antibody titres in all infants- an increase from only 18% seroconversion following the birth dose. This is in accordance with similar findings previously reported, where completion of the HepB vaccination schedule resulted in only moderate increases in HepB specific cellular responses, as compared to responses following a single dose, whilst antibody responses were significantly enhanced by the administration of several vaccine doses (11). Thus, we conclude that the cellular immune response may persist with the help of additional vaccine doses, but is not significantly enhanced by such, in contrast to what is seen with HepB humoral immunity.

In response to BCG stimulation, polyfunctional cells also increased in frequency when comparing D30 to D128, despite BCG being given as a single dose in the first week of life. As a result of immune maturation, infant responses to non-specific stimulation increase with age (24). This, together with the replicative nature of BCG likely explains the persistence and slight enhancement of BCG specific responses at D128 as compared to D30. Indeed, T-cell responses persisting long after BCG vaccination and, in some cases, increasing with age, has been demonstrated in both infants (24) and older subjects (25).

### Polyfunctional T- cells and correlation with antibody

We found that polyfunctional CD4^+^ T-cells correlated with anti-HBs antibody titres in neonates at D30 and D128, whereas no such correlation was seen between antibody titres and T-cells expressing only one marker, further implicating polyfunctional cells in enhancing the specific immune response to HepB vaccination.

A correlation between HepB specific CD4^+^ T-cell responses and antibody titres is supported by several previous studies: adults (26–28) and infants (17) with low antibody responses tend to show low or absent proliferation and cytokine production from T-cells, whereas strong T-cell responses have been measured in individuals with high antibody titres. Similarly, at D30 we detected higher frequencies of polyfunctional CD4^+^ T-cells in infants with HBsAb titres >10IU/l compared to those with titres below this threshold, but further analysis using this method was not feasible due to lack of heterogeneity in antibody titres measured. Instead, we performed Spearman’s correlation analysis between HBsAb titres and frequencies of HepB specific CD4^+^ T-cells and demonstrated a positive relationship between polyfunctional cells and antibody titres at both D30 and D128.

Although T-cell and antibody responses tend to correlate in strength, some studies have shown that T-cell responses are maintained even as antibody titres wane below levels of protection (5, 6, 8). In accordance with this, we did detect a substantial proportion of HepB specific CD4^+^ T-cells in infants with antibody titres below 10IU/l at D30. Considering that a single dose of HepB at birth is effective in protecting from neonatal infection, it is conceivable that activated CD4^+^ T-cells contribute to initial immunity, prior to development of protective antibody titres, though conclusively proving their involvement would require further functional studies, beyond the expression of activation markers.

Our results indicate that CD4^+^ T-cells may contribute to initial immunity, prior to the development of protective antibody titres, though further studies, in particular in serological non-responders, are needed to conclusively assign a role for CD4^+^ T-cells in neonatal protection to HBV.

### Strengths and limitations

To our knowledge, this is the first study to examine the cellular immune response to a single dose of HepB vaccine in such a large sample of infants. Our initial sample size of a total of 540 infants coupled with strict, pre-defined sample quality criteria, increases data reliability is a major strength of the current study. Limitations include a lack of a non-BCG group, meaning interpretations with regards to any enhancing effect of BCG on unrelated antigens was not possible. Furthermore, to ensure high cell viability and sufficient cell numbers in small-volume samples, PBMCs for detecting CD4^+^ T cell response at D30 and D128 were stained and acquired fresh over the 2-year sampling period. This did not allow for the synchronous analysis of matched pre and post vaccination samples, which is possible when using cryopreserved PBMCs. Although run-to-run variation was minimized by the use of calibration beads, it cannot be excluded that variation was introduced from one week to the next, though the large samples size somewhat mitigates this concern.

## Data availability statement

The datasets presented in this study can be found in online repositories. The names of the repository/repositories and accession number(s) can be found below: ImmPort (https://www.immport.org) study accession SDY1538.

## Ethics statement

The studies involving human participants were reviewed and approved by Gambia Government/Medical Research Council Unit The Gambia at London School of Hygiene and Tropical Medicine committee and Boston Children’s Hospital Institutional Review Board. Written informed consent to participate in this study was provided by the participants’ legal guardian/next of kin.

## Author contributions

Study conceptualization and funding acquisition: BK, OL, TK, AM. Methodology: BK, TK, RB-O, AM, KS, OI, GL-R, GS-S, AD, JS. Data analysis: JS, AD, JD-A, SV, SR, AO. Preparation of original draft: JS, AD. Editing: BK, TK, RB-O, AM. Finalizing manuscript: JS. All authors contributed to the article and approved the submitted version.

## Funding

This study was primarily funded by a grant received from the U.S. National Institute of Health (NIH); Grant number: NIAIDU19AI118608. BK, JS, AD, OI were supported by a UKRI-MRC core grant ref MC_UP_A900/1122. Additional funding was provided by the Precision Vaccines Program, supported in part by the Department of Paediatrics and Chief Scientific Office of Boston Children’s Hospital. These funders are not involved in study design, collection, management or analysis and interpretation of data, or publication of output.

## Acknowledgments

We thank the research lab, field team and research support team at MRC unit The Gambia, including: Modou Lamin Fofana, Aru-Kumba Baldeh, Adam Jeng-Barry, Sainabou Drammeh, Abdoulie Njie, Alansana Bah, Mamadou Bah, Lamin Sillah, Saikou Keita, Awa Badjan, Ndey Amie Njie, Fatoumata Fofana, Fatoumata Cole, Abdoulie Camara, Masanneh Ceesay, Assan Sanyang, Kahdijatou Jallow, Abdoulie E Jallow, Samba Jallow, Khadijatou Bah, Omar Minteh, Michelle John, Isatou Sanneh, Awa Keita, Abdoulie Camara, Ebrima Trawally, Elizabeth Njilan Johnson and Banjulinding Health Centre staffs for their tireless support and help and the parents for agreeing to enroll their newborns into this study.

## Conflict of interest

The authors declare that the research was conducted in the absence of any commercial or financial relationships that could be construed as a potential conflict of interest.

## Publisher’s note

All claims expressed in this article are solely those of the authors and do not necessarily represent those of their affiliated organizations, or those of the publisher, the editors and the reviewers. Any product that may be evaluated in this article, or claim that may be made by its manufacturer, is not guaranteed or endorsed by the publisher.
