# Supplementary material for: A single birth dose of Hepatitis B vaccine induces polyfunctional CD4+ T helper cells
Source: Front Immunol. 2022 Nov 8;13:1043375. doi: 10.3389/fimmu.2022.1043375 (PMC9681035; doi:10.3389/fimmu.2022.1043375)
Supplement: Supplementary file 1 [file DataSheet_1.docx]

Supplementary Material

# Supplementary Tables

**Supplementary Table 1.** Dilutions of antigens for stimulation of PBMCs

| **ANTIGEN** | **STOCK** | **CONCENTRATION** | **FINAL CONC. FOR STIMULATION** |
| --- | --- | --- | --- |
| HBsAg | Frozen aliquots | 5mg/ml | 1µg/ml |
| SEB | Frozen aliquots | 1 mg/ml | 1µg/ml |
| BCG vaccine | Reconstituted in 1ml sodium chloride (vaccine diluent) | 2-8x10^6^ CFU/ml | 10-40x10^4^ CFU/ml |

**Supplementary Table 2.** Conjugated antibodies and markers used to stain PBMCs

| MARKER_  FLUORO-  CHROME | DESCRIPTION | SOURCE | CAT # | CLONE | DILUTION |
| --- | --- | --- | --- | --- | --- |
| LIVE/DEAD^TM^ fixable red stain, 488nm | Dye allows discrimination of live and dead cells | Invitrogen | L23102 | N/A | 1/200 |
| CD4_V450 | Mouse anti-human CD4 antibody, used to detect CD4+ T-cells | BD | 651849 | SK3 | 1/50 |
| CD3_V500 | Mouse anti-human CD3 antibody, used to detect TCR expressing cells | BD | 652896 | SK7 | 1/50 |
| CD154_APC-Cy7 | Mouse anti-human CD154 antibody, used to detect antigen activated CD4 T-cells | Biolegend | 310822 | 24-31 | 1/100 |
| IL-2_APC | Rat anti-human Il-2 antibody, used to detect IL-2 producing CD4 T-cells | Biolegend | 500310 | MQ1-17H12 | 1/100 |
| TNFa_AF700 | Mouse anti-human TNFa antibody, used to detect TNFa producing CD4 T-cells | BD | 557996 | Mab11 | 1/200 |
| CD45RA_FITC | Mouse anti-human CD45RA antibody, used to detect Naïve CD4 T-cells | Invitrogen | 11-0458-42 | HI100 | 1/25 |
| CD27_PE-Cy7 | Mouse anti-human CD27 antibody, used in combination with CCR7 and CD45RA to differentiate T-cell memory phenotypes | e-bioscience | 25-0279-42 | O323 | 1/50 |
| CCR7_PE | Mouse anti-human CCR7 antibody, used in combination with CD27 and CD45RA to differentiate T-cell memory phenotypes | R&D | FAB197P-100 | 150503 | 1/12,5 |

| **Supplementary Table 3.** Sample quality exclusion criteria applied to CMI data | | |
| --- | --- | --- |
| **CRITERIA** | **DESCRIPTION** | **ACTION** |
| Low cell counts | Acquisition of few events result in unacceptably low cell counts in antigen specific cell populations | Exclude samples with <50000 CD4+ cells  or <10000 for SEB stimulated samples |
| Viability | Poor cell viability may create biases in cell population frequencies | Exclude samples with viability <90% |
| Poor flow | Choppy flow rate during acquisition may create biases in cell population frequencies | Exclude samples where <50% of events are in “time”gate |
| Protocol deviation | Samples with accidental antibody omission, stimulation error, mislabeling, contamination or other deviations | Evaluate protocol deviation comments. Exclude if appropriate |

**
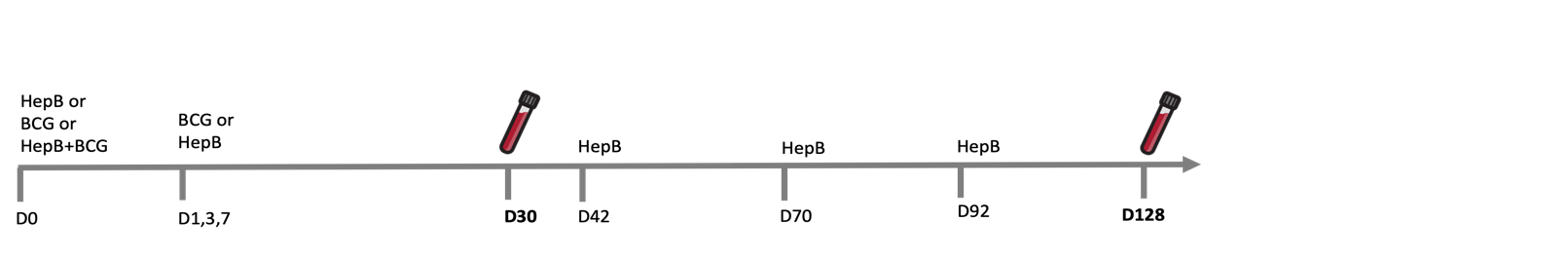
Supplementary Table 4.** vaccination schedule and demographics of study population

|  | | | | |
| --- | --- | --- | --- | --- |
| **INFANTS (n = 365)** | **TOTAL** | **MALE** | | **FEMALE** |
| **Number of subjects included** | 365 (100%) | 186 (51%) | | 179 (49%) |
| **Weight (g)** |  |  | |  |
| Weight birth | 3178 +/- 413 | 3239 +/- 432 | | 3114 +/-384 |
| Weight D30 | 4202 +/- 557 | 4320 +/- 587 | | 4078 +/- 496 |
| Weight D128 | 6670 +/- 866 | 6923 +/- 814 | | 6406 +/- 840 |
| **Age (days)** |  |  | |  |
| Age  Age at sampling D30 | 31 +/- 2 | 31 +/- 2 | | 31 +/- 2 |
| Age at sampling D128 | 127 +/- 4 | 127 +/- 4 | | 127 +/- 4 |
| **ADULT DONORS (n = 3)** | **AGE** | **SAMPLES** |  | |
| Donor 1 (male) | 39 | 42 | |  |
| Donor 2 (male) | 36 | 5 | |  |
| Donor 3 (male) | 29 | 26 | |  |

# Supplementary Figures


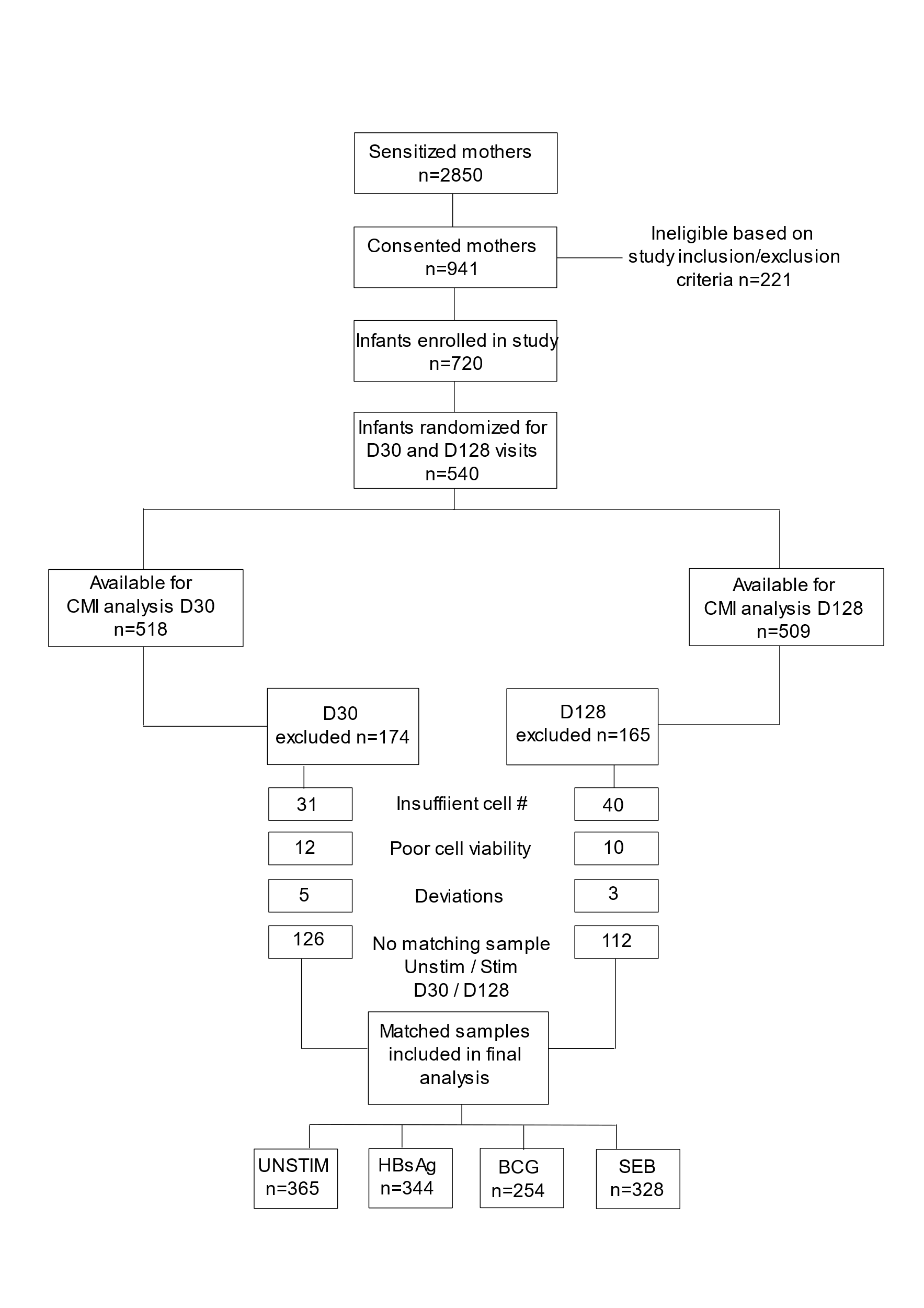


**Supplementary Figure 1. sample workflow for assessment of activated CD4^+^ T-cells.** Samples for assessment of CD4^+^ T-cell activation were acquired from a total of 518 (D30) and 509 (D128) day old infants. Pre-defined quality control criteria, including thresholds for CD4^+^ cell numbers and viability were applied. Protocol deviations were assessed and coded for exclusion or no exclusion. Samples from each study participant were matched to ensure availability of the corresponding unstimulated sample, as well as the presence of both blood draws (D30 and D128), leaving a total of 344, 254 and 328 matched samples stimulated with HBsAg, BCG and SEB respectively. Inclusion of BCG stimulation commenced ~2,5 months after initial study start, explaining the lower number of BCG stimulated samples.


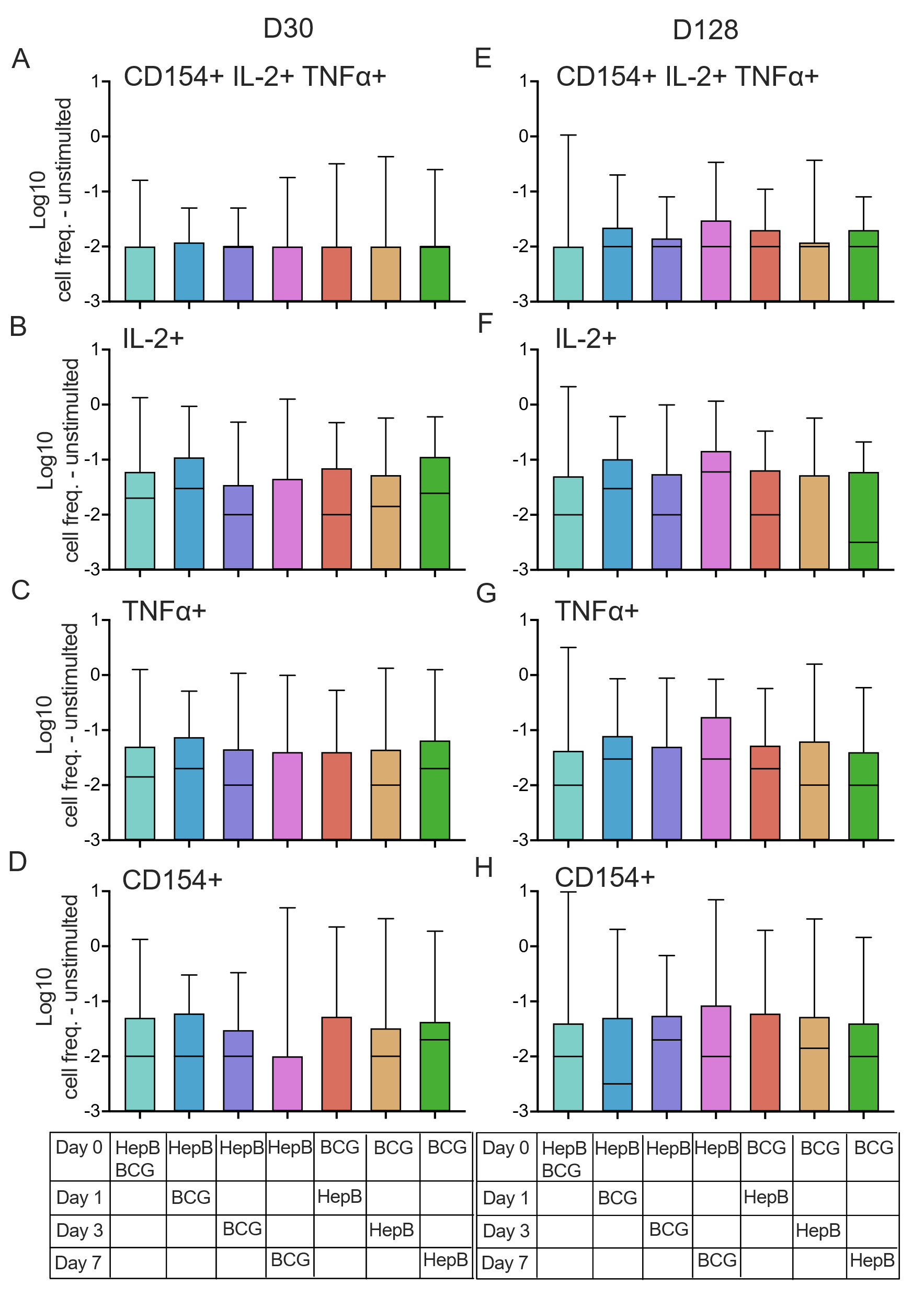


**Supplementary Figure 2: Lack of effect of BCG vaccine administration on HepB specific T-cell activation.** Infants received either HepB, BCG or both at birth (Day 0) and the omitted vaccine was delivered either on Day 1, -3, or -7. PBMCs were collected on D30 **(A-D)** and D128 **(E-H)** and stimulated with HBsAg for 18hrs after which CD4^+^ T-cell activation was measured. Activation induced by HBsAg was assessed via intracellular staining of CD154, IL-2 and TNF𝛼. Cell frequencies were analyzed based on expression of all three markers **(A,E)** or total expression of one marker **(B-D, F-H)**. Background activation was removed by subtracting values from unstimulated samples. Zero and negative values were imputed to 0.001 to enable Log transformation. Data are expressed as Log10 of CD4^+^ T-cell frequencies. Kruskal-Wallis with Dunn’s post-test was used to compare groups. All comparisons were non-significant (ns).

**Supplementary Figure 3: HepB specific CD4^+^ T-cell responses stratified by HBsAb titres.**

Infants were grouped based on their anti-HBsAb titres and cell frequencies of CD4^+^ polyfunctional T-cells **(A,D)** or frequencies based on total expression of IL-2 **(B,E)**, TNF𝛼 **(C,F)** or CD154 **(D,G)** were plotted against their matched antibody titres. Background CD4^+^ T-cell activation was removed by subtracting values from unstimulated matched samples. The proportion of infants with CD4^+^ T-cell frequencies above 0 after the subtraction of background is given below each group.

Only infants with undetectable HepB-specific antibody titres at birth were included. Kruskal-Wallis with Dunn’s post-test, *p < 0,05 ; **p < 0,01 ; *** p <0,005 ; **** p <0,001 ; ns = not significant.
